# Graded Exercise Therapy and Cognitive Behavioral Therapy for Fatigue in Patients With Breast Cancer: Protocol for a Randomized Controlled Pilot Study

**DOI:** 10.2196/91330

**Published:** 2026-08-07

**Authors:** Joanna Rui En Fong, Pei Ling Tan, Michelle Tian Nee Chow, Whee Sze Ong, Irene Teo, Ryan Shea Ying Cong Tan, Mothi Babu Ramalingam

**Affiliations:** 1 Rehabilitation Medicine Senior Residency Program, SingHealth, Outram Road, Singapore, 169608, Singapore, 65 62223322; 2 Department of Rehabilitation Medicine, Singapore General Hospital, Singapore, Singapore; 3 Division of Medical Oncology, National Cancer Centre Singapore, Singapore, Singapore; 4 Division of Clinical Trials and Epidemiological Sciences, National Cancer Centre Singapore, Singapore, Singapore; 5 Department of Psychosocial Oncology, National Cancer Centre Singapore, Singapore, Singapore

**Keywords:** breast neoplasms, fatigue, exercise therapy, cognitive behavioral therapy, telerehabilitation

## Abstract

**Background:**

Fatigue is a common debilitating symptom of breast cancer (BC), and its treatment may result in a significant symptom burden and affect adherence to treatment. Graded exercise therapy (GET) and cognitive behavioral therapy (CBT) have separately been shown in previous studies to be beneficial for the management of cancer-related fatigue.

**Objective:**

This study’s coprimary aims are to assess the feasibility and acceptability of combining GET and CBT for the treatment of fatigue in patients with BC in Singapore. The secondary aims are to generate preliminary efficacy estimates of the combination therapy.

**Methods:**

In this randomized controlled pilot study, a total of 100 female patients with BC, with a self-reported rating of at least moderate fatigue (One-Item Fatigue Scale score ≥4), will be recruited and randomized in a 1:1 ratio to undergo a combination of GET and CBT vs GET alone (standard of care). This will include a primary cohort of 90 patients with stage I to III BC who have completed surgery and adjuvant chemotherapy (if indicated), and an exploratory cohort of 10 patients with stage IV BC undergoing systemic therapy. Acceptability will be measured using the Client Satisfaction Questionnaire, including items on cultural sensitivity. Feasibility will be measured by participant uptake, adherence to sessions, and willingness to pay for therapy sessions. Efficacy will be assessed based on quantitative measures of fatigue, treatment adherence, quality of life, and physical and functional outcomes.

**Results:**

Recruitment of participants commenced on July 14, 2025, and is projected to be completed by December 31, 2026. The primary completion date is expected to be April 30, 2027, and publication of the final results is expected by the end of 2027.

**Conclusions:**

This study will provide evidence on whether a combination of GET and CBT is feasible and acceptable for the treatment of fatigue in patients with BC in Singapore. It supports the refinement of evidence-based fatigue management guidelines and improvements in BC survivorship care.

## Introduction

Since global estimates of cancer were first published in 1984, breast cancer (BC) has remained the most frequently diagnosed cancer in women [1,2]. In 2022, there were an estimated 2.3 million new cases and 670,000 deaths from BC, with these figures projected to increase by 38% and 68%, respectively, by 2050 [3]. Systemic therapies such as chemotherapy, targeted therapies, and endocrine therapies have significantly improved overall survival in BC [4]. However, these treatments are associated with a significant symptom burden that can negatively impact patients’ quality of life (QoL) and ability to adhere to the treatment regimen, with consequent detrimental effects on treatment and survival outcomes [5].

Fatigue is a commonly reported symptom after BC treatment. In a review of fatigue in patients with BC, 66% reported the presence of some degree of fatigue, with one-third reporting that fatigue constituted an important problem [6]. For example, in the pooled analyses of the PALOMA (Palbociclib: Ongoing Trials in the Management of Breast Cancer) trials, which established the role of the cyclin-dependent kinase 4/6 inhibitor palbociclib in BC treatment, new-onset fatigue incidence rates were 23.9%, with an incidence of severe fatigue of 14.8% [7]. A separate meta-analysis found that patients who were on cyclin-dependent kinase 4/6 inhibitor–based regimens had a significant increase in all grades of fatigue, and a relative risk of 3.06 (95% CI 1.41‐6.61; *P*=.004) for high-grade fatigue [8].

As a result of fatigue and other contributory factors, suboptimal adherence to oral treatments for BC is a well-documented problem, with discontinuation ranging from 31% to 73% by 5 years of treatment [9]. Adverse events are closely related to patient adherence and persistence. A survey of patients’ experiences of cancer treatment, including patients with BC, found that 10.1% of poor adherence was directly attributed to fatigue; 27% reported severe fatigue, with 19% confirming poor treatment adherence [10]. In patients with BC receiving endocrine treatment, those with nonadherence and nonpersistence had significantly reduced event-free survival, with hazard ratios ranging from 1.39 (95% CI 1.07-1.53) to 2.44 (95% CI 1.89-3.14), and also negatively impacted overall survival, with hazard ratios ranging from 1.26 (95% CI 1.11-1.43) to 2.18 (95% CI 1.99-2.39) [11].

Current evidence supports the inclusion of exercise to manage cancer-related fatigue (CRF) during and after the completion of cancer treatment as part of standard care [12]. However, the delivery of these interventions may present challenges with patient adherence to the designed interventions. Cognitive behavioral therapy (CBT) is an evidence-based supportive intervention that has been shown to improve symptom burden and QoL in patients with BC [13]. CBT could potentially address both the cognitive and behavioral factors that could impede motivation for exercise. Thus, the addition of CBT to graded exercise therapy (GET) should have a synergistic effect in the management of fatigue in patients with BC [14].

This pilot study evaluates the combination of GET and CBT for the treatment of fatigue in patients with BC in Singapore.

## Methods

### Study Design

This is a randomized controlled, nonblinded, pilot intervention study that will enroll 100 female patients with BC with reported fatigue to receive either a combination of GET and CBT or GET alone.

### Ethical Considerations

The study will be conducted in accordance with the ethical principles that have their origin in the Declaration of Helsinki and that are consistent with Good Clinical Practice and the applicable regulatory requirements. Ethical approval was obtained from the SingHealth Central Institutional Review Board (protocol number 2025/0016). Written informed consent was obtained from all participants involved in the study.

Zoom, a cloud-based videoconferencing platform, will be used as the platform to facilitate GET and CBT teleconsultations. No data will be collected or stored using this platform. Access to Zoom sessions will be restricted to authorized members of the study team.

FormSG, a Singapore government platform for the creation of secure digital forms, will be used to collect survey responses, including both personal data (eg, name and contact details) and research data (eg, survey responses). The FormSG account and surveys will be protected by a username and password. All data collected will be stored on encrypted storage devices secured under lock and key or on a hospital-approved secure drive located within the hospital firewall. Access to the data will be limited to authorized members of the study team. Research data will be identifiable only by patient ID. The file linking patient IDs to personal identifiers will be stored separately from the research data. Participants’ privacy will be safeguarded, and personal data will not be disclosed to any third party.

### Specific Aims

The coprimary aims are to evaluate: (1) acceptability of GET and CBT, using the Client Satisfaction Questionnaire-8 (CSQ-8), including items on cultural sensitivity; and (2) feasibility of GET and CBT as indicated by participant uptake and adherence to sessions and participants’ willingness to pay for therapy sessions.

We hypothesize that at least 70% of patients with BC will find the combination of GET and CBT to be acceptable and will adhere to their therapy sessions.

The secondary aims include the following:

1. Assessment of preliminary efficacy in reducing fatigue using the Functional Assessment of Chronic Illness Therapy–Fatigue (FACIT-F) subscale
2. Evaluation of adherence to BC treatments using self-reported adherence data and estimations from dispensed medication possession ratios
3. Evaluation of QoL using the Functional Assessment of Cancer Therapy–General (FACT-G) questionnaire
4. Assessment of physical activity and functional outcomes using the Godin-Shephard Leisure-Time Physical Activity Questionnaire (GLTPAQ), 6-minute walk test (6MWT), handgrip strength (HGS), and 5-times sit-to-stand test

### Eligibility Screening and Recruitment

The study cohort will include a primary group of 90 patients with nonmetastatic BC (stage I-III) and an exploratory group of 10 patients with stage IV BC. The exploratory group will be combined with the primary cohort to generate outcome estimates in a broader population with BC to facilitate future study planning. Textbox 1 details the inclusion and exclusion criteria for both the primary and exploratory groups.

Textbox 1. **Inclusion and exclusion criteria.**

**Inclusion criteria**

- Primary group
  - Diagnosed with stage I to III breast cancer (BC), regardless of hormone receptor status or human epidermal growth factor receptor 2 status
  - Completed surgery and any adjuvant chemotherapy (if indicated)
- Exploratory group
  - Diagnosed with stage IV BC
  - Currently receiving systemic therapy (including endocrine therapy, chemotherapy, targeted therapy, or immunotherapy)

- For both groups
  - Aged 21 years or older
  - Experiencing fatigue, with a One-Item Fatigue Scale score ≥4 (at least moderate fatigue)
  - Able to read and communicate in English
  - Willing to provide informed consent for study participation

**Exclusion criteria**

- Pregnant or lactating
- Presence of dementia or major psychiatric disease
- Deemed medically unsuitable by the medical team for low- to moderate-intensity exercise
- Unable or unwilling to participate in video counseling sessions
- Presence of uncontrolled or undiagnosed pain
- Low blood counts: hemoglobin <8.0 g/dL, platelets <50 × 10^9^/L, absolute neutrophil count <1 × 10^9^/L, potassium <3 mmol/L, or sodium <130 mmol/L
- Presence of cardiovascular disease, including reduced left ventricular ejection fraction <30%, uncontrolled arrhythmias, severe coronary artery disease, severe valvular heart disease, or uncontrolled hypertension that has not been cleared by a cardiologist
- Presence of an active infection
- Already engaged in moderate- to vigorous-intensity physical activity

Study investigators and research assistants will screen participants based on the above inclusion and exclusion criteria and will approach eligible participants during their clinic appointments or via telephone to introduce the study. Interested participants will be screened for fatigue using the One-Item Fatigue Scale score (“How would you rate your fatigue on a scale of 0 to 10, with 0 being ‘no fatigue’ and 10 being ‘the worst possible fatigue’?”), with a score of 4 or more indicating at least moderate fatigue. This one-item scale has demonstrated convergent validity with other measures of fatigue and has been shown to be able to identify cases of fatigue meeting criteria on the FACIT-F scale [15].

### Setting

Participants will be recruited from multiple sites across Singapore: Singapore General Hospital, a tertiary hospital, the National Cancer Centre Singapore, the national oncology specialty center based at the Outram Campus, and National Cancer Centre Singapore satellite clinics at Sengkang General Hospital and Changi General Hospital.

### Experimental Design

**Figure 1.**
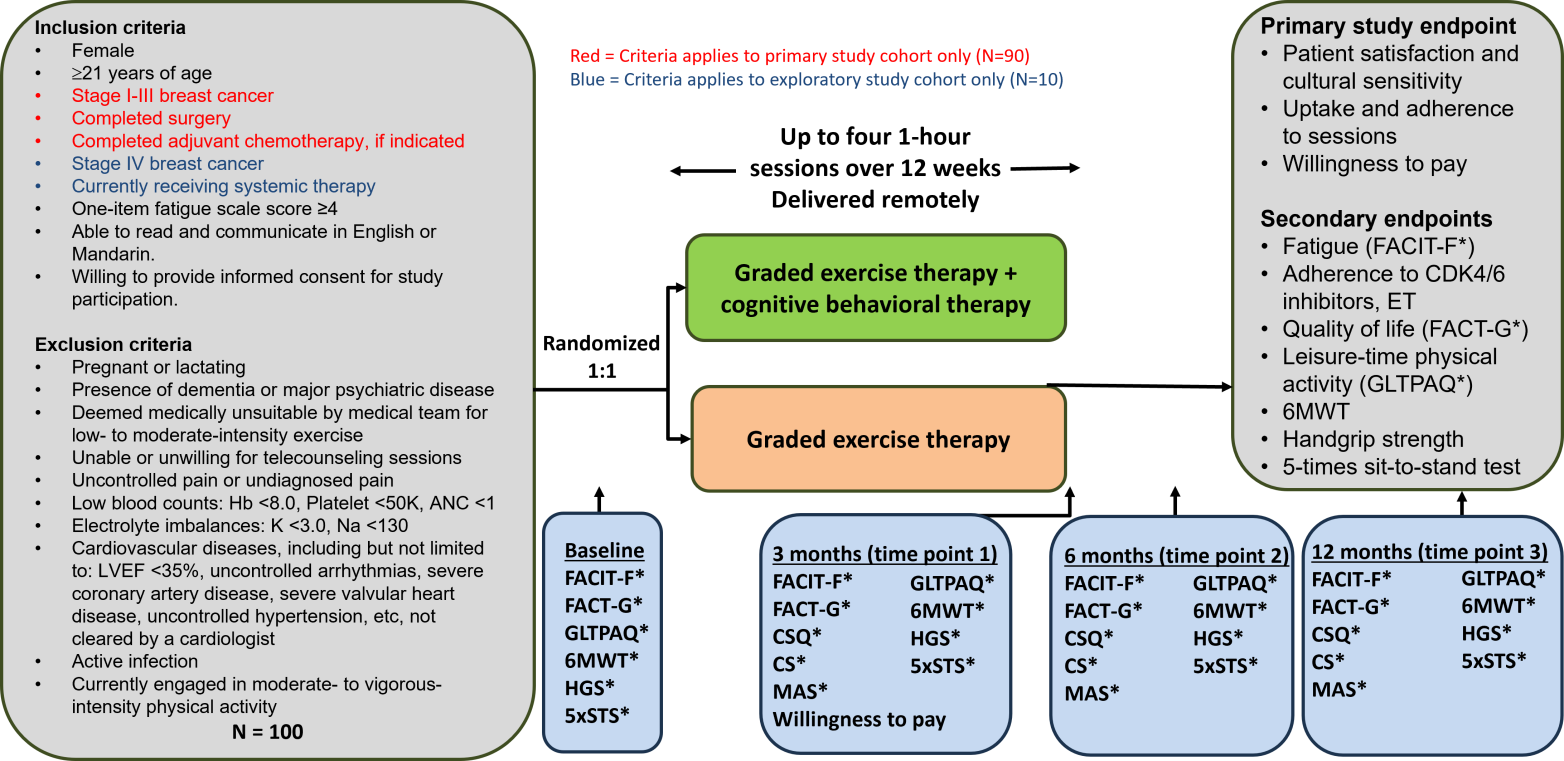
Workflow of study participants from initial screening, through delivery of the graded exercise therapy and cognitive behavioral therapy interventions to subsequent follow-up. At the 6- and 12-month time points, participants will also be asked about adherence to exercise and cognitive behavioral therapy strategies. 5×STS: 5-times sit-to-stand test; 6MWT: 6-minute walk test; ANC: absolute neutrophil count; CDK4/6: cyclin-dependent kinase 4/6; CS: cultural sensitivity assessment items; CSQ: Client Satisfaction Questionnaire-8; ET: endocrine therapy; FACIT-F: Functional Assessment of Chronic Illness Therapy-Fatigue; FACT-G: Functional Assessment of Cancer Therapy–General; GLTPAQ: Godin-Shephard Leisure-Time Physical Activity Questionnaire; Hb: hemoglobin; HGS: handgrip strength; LVEF: left ventricular ejection fraction; MAS: Medication Adherence Scale.

Recruited participants will be randomized via a web-based system to either the GET-only arm or the GET plus CBT arm in a 1:1 ratio using a randomized permuted block method, with stratification by study site. The randomization list is generated by the study statistician, and block sizes are kept unknown to all other study personnel. The study schema is shown in Figure 1. Both GET and CBT sessions will be delivered remotely by trained rehabilitation physicians and clinical psychologists, respectively, via the Zoom videoconferencing platform.

Exercise therapy will consist of a self-supervised, individualized exercise program prescribed by the rehabilitation physician. This graded exercise program consists of both aerobic (eg, brisk walking and cycling) and resistance exercises, customized to the participant’s pre-existing medical conditions and preferences. Exercise will be prescribed in a graded manner across 3 phases, at weeks 0, 4, and 8 (Table 1). The Modified Borg Rating of Perceived Exertion scale will be introduced to the participant, where the desired intensity of exercises is moderate, with a Rating of Perceived Exertion of 4‐6.

**Table 1.** Graded exercise program (3 phases).

Phase	Weeks	Aerobic exercises	Resistance exercises
1. Foundation	0-4	3 times per week, 20-25 min, low- to moderate-intensity (RPE^a^ 3‐5)	2 times per week, at least 3 exercises. 1-2 sets of 8-12 repetitions of each exercise
2. Build up	5-8	3-4 times per week, 30 min, moderate intensity (RPE 4‐6)	2-3 times per week, at least 5 exercises. 2-3 sets of 8-12 repetitions of each exercise
3. Strength and endurance	9-12	4 times per week, 35-45 min, moderate intensity (RPE 5‐6)	2-3 times per week, at least 7 exercises. 2-3 sets of 8-12 repetitions of each exercise

a RPE: rating of perceived exertion.

At the first video consultation session, the rehabilitation physician will go through the exercise booklet in detail with each participant. A study research assistant will conduct weekly phone calls to check on the participants’ adherence to and compliance with the prescribed exercises. Over the 3 subsequent follow-up video consultation sessions (weeks 4, 8, and 12), the rehabilitation physician will review the exercises with the participants and implement modifications, if necessary, based on feedback from the participants. The type of aerobic exercise and resistance exercise will be tailored to the participant’s preference and guided by the rehabilitation physician.

Adverse event monitoring for GET is conducted via the following three methods:

1. Weekly phone calls are made by the study coordinator to check for the onset of any new symptoms, distress, or adverse events. Participants are advised to stop the exercises if they experience excessive fatigue (score >7 on the One-Item Fatigue Scale) or develop new and/or unusual pain, chest discomfort, nausea, severe dyspnea, or palpitations while engaging in exercise.
2. Monthly video consultations with rehabilitation physicians.
3. An exercise diary with provisions for participants to record any adverse events.

Any adverse events are also reported to the clinical investigators in the study team for further follow-up and a decision to continue with the study.

The CBT intervention will be delivered by trained clinical psychologists or psychology trainees under the supervision of a clinical psychologist across four 1-hour intervention sessions, delivered in English via telehealth within 12 weeks. This intervention will have 4 major components that have been adapted from previous randomized controlled trials (RCTs) evaluating the use of CBT in managing fatigue in BC [13,16,17].

The session structure and outline are as follows:

- Session 1: psychoeducation on CRF, establishing personalized value-driven goals, the use of a fatigue tracking diary, and relaxation training.
- Session 2: behavioral skills training for symptom management of fatigue through energy conservation, activity pacing, and goal setting. We will also have a time allowance to discuss related issues such as sleep disturbance, mood, and cognitive challenges.
- Session 3: psychoeducation on the cognitive behavioral perspective using the ABCD (A: activating event, B: beliefs, C: consequences, and D: dispute) model, followed by cognitive strategies to manage worries.
- Session 4: strategies to maximize social support and change dynamics within the family. Consequently, we will summarize the skills learned in the last 4 sessions.

The CBT intervention will be tailored for local use. For example, adaptation of materials for a low reading level, inclusion of minimal writing exercises, and the use of examples and discussions surrounding social roles that have been amended to fit the local context. Summaries of the information for each session will be provided for participants’ reference, and they will also be asked to practice the skills introduced in the intervention after each session. The first 10 to 20 minutes at the start of each session are allocated to check on participants’ welfare and review their application of previously taught skills. Participants who indicate high distress will be assessed for safety, triaged, and referred to another mental health care professional for additional support if appropriate. Feedback will be qualitatively obtained from participants at the end of the fourth session, and any technical difficulties will be documented in the relevant consultation notes if any challenges were observed during the session.

The administration of the sessions is protocolized using therapist manuals, and prior to commencing sessions, therapists receive standardized training. Therapists are recorded during the sessions, with the content compared against a checklist to ensure that all relevant points have been covered during each session. Therapists also complete a form to indicate any deviation from the protocol, such as participants declining a certain activity or a longer time being taken than usual due to technical glitches.

Outcome measures will be assessed at 4 time points as illustrated in Figure 1: baseline, 3 months (time point 1), 6 months (time point 2), and 12 months (time point 3). At baseline, demographic information will also be collected, including reproductive history, education, marital status, and employment history, using a questionnaire adapted from a previous multiethnic cohort phase 2 study in the Singaporean population [18]. Participants will be evaluated for their perception of self-efficacy, measured using the Exercise Self-Efficacy Scale [19], and will also be screened for depression and anxiety symptoms using the 10-item Center for Epidemiologic Studies Depression Scale [20,21] and the Generalized Anxiety Disorder-7 scale [22], respectively.

### Measures

#### Primary Endpoints

The primary acceptability endpoint is defined as the percentage of participants who score at least 24 (out of a maximum of 32) on the CSQ-8 survey at 3 months. The primary feasibility endpoint is defined as the percentage of participants who are adherent to their therapy sessions (attending at least 3 out of 4 sessions for GET and CBT each).

#### CSQ-8

The CSQ-8 is an 8-item questionnaire that was developed as a brief global measure of client satisfaction and was abbreviated from the longer 18-item version of CSQ-8 [23]. The CSQ-8 was shown to have excellent performance in comparison with the original CSQ-18 [24]. In this study, the CSQ-8 will be used, along with additional questions about whether the program helped participants to better understand the overall experience of BC, and which version of CBT materials the participants found useful. The CSQ-8 score ranges from 8 to 32, with higher scores indicating greater satisfaction.

#### Cultural Sensitivity

Cultural sensitivity, which is the perceived cultural relevance of the program to the participant’s individual culture, will be assessed using 4 items adapted from the Cultural Sensitivity Assessment Tool [25]. Participants will respond to each item on a 4-point scale (ranging from “strongly disagree” to “strongly agree”). Scores range from 4 to 16, with higher scores indicating greater satisfaction and cultural sensitivity. This measurement tool has been successfully used in prior Singapore-based studies [26-28].

#### Uptake and Adherence

Participant uptake will be assessed based on the percentage of randomized patients who attended at least 1 session of their allocated intervention and the percentage of randomized patients who completed their allocated intervention.

Participant adherence during the study intervention period will be assessed based on the number of sessions of their allocated intervention attended by the patients. To assess participant adherence during the follow-up period, the following questions will be used:

1. For adherence to GET: “In the past 3 months, how often did you do your exercises as prescribed?”
2. For adherence to CBT: “In the past 3 months, how often did you use any of the CBT strategies to manage your fatigue?”

Participants will respond on a 5-point scale (5=“all of the time,” 100%; 4=“nearly all of the time,” 90%; 3=“most of the time,” 75%; 2=“about half the time,” 50%; and 1=“less than half of the time,” <50%). Nonadherence is defined as those who answer 3 (“most of the time,” 75% or less).

#### Willingness to Pay

Participants will be asked about their willingness to pay for the program, pegged to 5 different prices that reflect the varying levels of government subsidy for the private and subsidized tiers available in public hospitals in Singapore. This information can be used for planning future subvention requests for GET and CBT, or for implementation of the program extending beyond the scope of this study.

#### Fatigue—FACIT-F

The 13-item FACIT-Fatigue subscale assesses self-reported fatigue and its impact on activities of daily living and function [29], and is scored on a 5-point Likert-type scale with reference to the preceding week. It has been validated in patients with cancer aged 18 years and older. The FACIT-F score ranges from 0 to 52, with higher scores indicating less fatigue.

#### Medication Adherence—Medication Adherence Scale

Self-reported medication adherence data and estimations from dispensed medication possession ratios will be used, following methodology from previous studies on medication adherence [30,31]. It will assess compliance with medications over the preceding 3 months. The primary measure item asks how often participants took their medication as prescribed over the preceding 3 months. Nonadherence is defined as 75% of the time or less.

#### QoL—FACT-G

The FACIT-General is a 27-item questionnaire designed to measure 4 domains of health-related QoL in patients with cancer, namely physical, social, emotional, and functional well-being [32]. It is scored on a 5-point Likert-type scale with a recall period over the preceding week. This questionnaire has been well validated in patients with cancer aged 18 years or older. The FACT-G total score ranges from 0 to 108, with higher scores indicating better QoL. The total score is broken down into 4 subscales: physical (0‐28), social (0‐28), emotional (0‐24), and functional well-being (0‐28).

#### Physical and Functional Outcomes

##### GLTPAQ

The GLTPAQ is a simple, self-reported questionnaire that is widely used to measure the amount of physical activity that a participant engages in during a typical 7-day period, with information gathered on the number of times one engaged in mild, moderate, and strenuous activity [33].

##### 6MWT

The 6MWT is a self-paced, submaximal exercise test measuring the distance a participant can walk within 6 minutes (in meters), assessing their aerobic capacity and endurance. The distance walked within 6 minutes can also be used to compare changes in functional capability [34].

##### Handgrip Strength

HGS, measured with a handheld dynamometer, provides a surrogate measure of overall muscle strength and physical function [35]. Measurement position is standardized, with the participant seated and their elbows at 90°, and measurements will be done in triplicate on both arms, with the highest value recorded (in kilograms).

##### 5-Times Sit-to-Stand Test

The 5-times sit-to-stand test evaluates lower limb strength and dynamic balance and provides an indication of the risk of falls in an individual [36]. The participant begins seated on a chair (without armrests or wheels), with arms folded across their chest. They will stand fully upright and sit down again with their back fully against the backrest, as quickly as possible. The time taken to complete 5 repetitions is recorded (in seconds), with a lower time indicating a better outcome.

### Study-Specific Exploratory Items

In addition to the validated outcome measures described above, participants will be asked to complete study-specific patient feedback items.

Close-ended items assessing the perceived helpfulness of the intervention:

1. “To what degree did this program help you to better understand the overall experience of breast or gynecological cancer?”
2. “To what degree did the program teach you skills that are helping you to better manage your symptoms?”

Responses will be recorded on a 4-point Likert scale, with higher scores indicating greater perceived helpfulness.

For participants assigned to the GET plus CBT arm, an additional close-ended categorical item will assess the use of CBT materials:

1. “Which version of the CBT handout did you find most useful?”

Participants will select 1 of the 4 response options: digital only, print only, both digital and print, or neither.

Two additional open-ended questions will invite participants to describe the most and least helpful parts of the program and provide suggestions for improvement.

### Expected Risks

There are no additional toxicities or adverse effects anticipated, given that participants who have consented to the study will continue to receive medical management in line with usual care, which includes exercise therapy by rehabilitation professionals. The CBT procedures in this study have been previously described without additional adverse effects. During the consent process, patients will be informed that their participation in this study is voluntary. Withdrawal from the study will not affect their treatment, care, or any benefits to which they are entitled. The consent process will be carried out in a quiet and conducive room as far as possible. Provisions for adverse event monitoring for both GET and CBT have been described above under the *Experimental Design* section. All participants may choose to withdraw from the study completely at any point in time.

As the study is considered minimal risk, the principal investigator is responsible for monitoring the data, assuring protocol compliance, and conducting safety reviews. During the review process, the principal investigator will evaluate whether the study should continue unchanged, require modification or amendment, or close to enrollment. A log of adverse events will be maintained by the study staff and reviewed on a regular basis by the principal investigator. Serious adverse events will be reported as per institutional review board guidance. Adverse events will be categorized and summarized by intervention arm using counts and proportions. The study protocol will be reviewed by the institutional review board. Protocol changes will be communicated to relevant parties via weekly team meetings and timely email communications.

### Statistical Methods

This study combines the A’Hern single-arm decision framework with a randomized 2-arm design. The single-arm decision framework is applied to the GET and CBT arms to evaluate the coprimary aims of feasibility and acceptability in the primary study cohort of patients with stage I-III BC. The randomized 2-arm design is adopted to estimate differences in preliminary efficacy estimates between the GET and CBT arms and the GET-only arm. Sample size is estimated based on the A’Hern single-arm decision framework. Under this framework, the combined GET and CBT will be considered feasible and acceptable if at least 70% of patients in the primary study cohort score at least 24 points on the CSQ-8 survey at 3 months and attend at least 3 out of 4 sessions for GET and CBT each. No formal hypothesis testing of between-arm differences will be undertaken, as the study is not powered for comparative efficacy analyses and will focus on generating preliminary estimates of effect.

The study team considers the combination of GET and CBT worthy of further investigation if at least 70% of eligible participants randomized to the GET plus CBT arm find the therapy acceptable and adhere to their therapy sessions. Conversely, the combination therapy will not be worthy of further investigation if the acceptability and feasibility rate is less than 50%. Based on the A’Hern design, the combination of GET and CBT will be worthy of further investigation with 80% power and a 5% significance level if at least 24 of 37 participants find the therapy acceptable and adhere to their therapy sessions. With a 1:1 allocation ratio in this randomized study, a total of 90 participants will be recruited, with 45 participants in each arm, after accounting for dropout. An exploratory study cohort of 10 patients with stage IV BC is included in this study, and the total sample size is 100 participants.

All analyses, except for safety data, will be based on an intention-to-treat approach. Safety data will be reported based on the actual intervention received by participants. Descriptive statistics will be used to summarize participants’ characteristics, the willingness-to-pay survey, safety data, and close-ended participant feedback items. Responses to open-ended questions will be analyzed using descriptive content analysis to identify common themes and illustrative examples. The primary feasibility endpoint will be estimated based on the exact method. The primary acceptability endpoint will be estimated based on a mixed effects logistic regression model. This model will include indicator variables for postbaseline assessment time points and study sites as fixed effects and patient-specific random intercepts. Continuous secondary outcome measures, including FACIT-F, Medication Adherence Scale, FACT-G, GLTPAQ, 6MWT, HGS, and 5-times sit-to-stand test, will be analyzed using mixed effects linear regression models for repeated measures. For each measure, the change in score between assessment time points within each arm and the difference in scores between the 2 arms at the various assessment time points will be estimated and reported with 95% CIs. Each mixed effects linear model will include study arm, indicator variables for postbaseline assessment time points, interaction terms between the time variables and study arm, and study sites as fixed effects and patient-specific random intercepts. Models estimating the difference in scores between the 2 arms at various assessment time points will be further adjusted for baseline score. The choice of the structure used to fit the covariance matrix of each mixed effects model will be assessed at the time of analysis. No imputation for missing data will be carried out, and all patients with nonmissing data at any one of the assessment time points will be included in the analysis. No adjustment for multiple comparisons of secondary outcome measures will be made.

Two sets of analyses will be conducted. The first set will be based on the primary study cohort, and the second set will be based on the combined primary and exploratory study cohorts. For the second set, no hypothesis testing will be conducted. The focus of this set is to generate outcome estimates in all patients with BC to facilitate future study planning.

## Results

Recruitment of participants commenced on July 14, 2025, and is projected to be completed by December 31, 2026. As of the end of April 2026, 48 participants had been recruited. Data analysis had not commenced at the time of submission of this research protocol, as data collection was still ongoing. The primary completion date is expected to be April 30, 2027, and publication of the final results is expected by the end of 2027.

## Discussion

### Trial Design and Rationale

This protocol, which adheres to the SPIRIT (Standard Protocol Items: Recommendations for Interventional Trials) guidelines (Checklist 1), describes the combination of 2 established therapies for fatigue management in patients with BC.

GET and CBT have been individually studied for the management of posttreatment fatigue in cancer survivors. Poort et al [13] found that a CBT intervention significantly reduced fatigue compared with usual care in advanced cancers, whereas the GET intervention resulted in a small improvement in fatigue. Sandler et al [14] further demonstrated that a combination of GET and CBT was beneficial for fatigue and functional outcomes in patients with breast or colon cancer who had posttreatment fatigue. This study compares the use of a combination of GET and CBT against GET alone for the management of fatigue in patients with BC in Singapore. Its coprimary aims are to establish the feasibility and acceptability of GET and CBT interventions in the local context, with secondary aims to generate preliminary efficacy estimates of the combination therapy in terms of fatigue, BC treatment adherence, QoL, and functional outcomes.

### Strengths and Limitations

The greatest strength of this study is the input from a multidisciplinary team, with expertise from oncological, psychological, and rehabilitation perspectives. This allows for a holistic approach to the management of fatigue, with complementary perspectives to formulate a well-rounded program. Second, the remotely delivered, patient-led, and individualized design of the program empowers participants to take ownership of their management. This may result in more efficient resource utilization and implementation at scale compared to traditional in-person delivery of rehabilitative and psychological therapies. Third, this detailed protocol serves to facilitate future training and dissemination to relevant health care personnel who provide care to patients presenting with CRF.

The main limitation that the team foresees would be technological challenges that may impede the delivery of GET and CBT interventions. These include factors such as Wi-Fi connectivity, access to appropriate technological equipment (eg, a device with a webcam), and sufficient technology literacy to operate them, which may be more significant in the lower educated or older populations. Another limitation is that this study is nonblinded and may be susceptible to various biases. Self-reported outcomes in the GET plus CBT arm can be distorted by patients who may report more positive outcomes due to their belief that CBT is effective in reducing their fatigue. To mitigate this expectation bias, objective outcome measures are used to supplement self-reported data for study endpoints, wherever feasible. For instance, adherence to BC treatments is estimated based on medication possession ratios, which are derived based on drug dispensing records, in addition to patients’ responses to the medication adherence survey form. To manage a patient’s expectations, it is highlighted in the patient information sheet that the investigators are not clear whether patients will derive benefit with the addition of CBT, given that there are limited studies on the effects of CBT on fatigue. To prevent performance bias, rehabilitation physicians prescribing exercise therapy are kept blinded to the study arm of each patient. To minimize detection bias, the recruiting research assistant who conducts all physical tests on patients will follow a standardized set of procedures. A further limitation is that participants in the GET and CBT arm receive additional therapist contact through CBT sessions, which is not provided in the GET-only arm. Consequently, any observed differences in outcomes between the 2 arms cannot be unequivocally attributed to the specific therapeutic components of CBT. A future definitive RCT could address this by incorporating an attention-control condition for the GET-only arm, thereby enabling clearer isolation of the specific effects of CBT from those related to increased therapist contact.

### Significance and Clinical Implications

Findings from this pilot study will inform the design of a future definitive RCT in several ways. Effect size estimates for fatigue and QoL will provide empirical data to support power calculations for the primary efficacy endpoint. Recruitment and attrition rates observed in the pilot will enable estimation of a realistic sample size for a fully powered trial. In addition, the study team will conduct a comprehensive review of all pilot findings to determine whether progression to a definitive RCT is warranted. The pilot study will be reported in accordance with the CONSORT (Consolidated Standards of Reporting Trials) extension for randomized pilot and feasibility trials.

### Conclusions

The combination of CBT and GET may potentially manage CRF in patients with BC more effectively than either therapy alone by addressing the dual burden of physiological fatigue and psychological resistance to exercise adherence. This pilot study provides actionable guidance in fatigue management, with implications for enhancing BC survivorship in Singapore.

## Supplementary material

10.2196/91330 Checklist 1 SPIRIT checklist.

## Abbreviations

6MWT: 6-minute walk test
BC: breast cancer
CBT: cognitive behavioral therapy
CONSORT: Consolidated Standards of Reporting Trials
CRF: cancer-related fatigue
CSQ-8: Client Satisfaction Questionnaire-8
FACIT-F: Functional Assessment of Chronic Illness Therapy–Fatigue
FACT-G: Functional Assessment of Cancer Therapy–General
GET: graded exercise therapy
GLTPAQ: Godin-Shephard leisure-time physical activity questionnaire
HGS: handgrip strength
PALOMA: Palbociclib: Ongoing Trials in the Management of Breast Cancer
QoL: quality of life
RCT: randomized controlled trial
SPIRIT: Standard Protocol Items: Recommendations for Interventional Trials
